# Epidemiological characteristics and incidence prediction of varicella from 2014 to 2023 in Chongqing, China

**DOI:** 10.3389/fpubh.2026.1722951

**Published:** 2026-02-19

**Authors:** Haomin Tang, Shuangyan Mao, Peiji Yang, Qingqing Fan, Dayong Xiao, Dan Deng

**Affiliations:** 1 College of Public Health, Chongqing Medical University, Chongqing, China; 2 Chongqing Municipal Center for Disease Control and Prevention, Chongqing, China

**Keywords:** epidemiological characteristics, LSTM, SARIMA, SARIMA-LSTM, time series predictive models, varicella

## Abstract

**Objective:**

To characterize the epidemiology of varicella in Chongqing during 2014–2023, and establish the most suitable prediction model for the varicella incidence trends in the city, providing scientific support for early warning of the varicella incidence trend, the formulation and the optimizing of precise varicella preventive strategies.

**Methods:**

Varicella reported cases in Chongqing during 2014–2023 were collected to characterize the epidemiology, all the varicella cases were sourced from the “Information management system for infectious disease reporting.” Seasonal autoregressive integrated moving average (SARIMA) model, long short-term memory (LSTM) model and SARIMA-LSTM hybrid models were established based on the surveillance data. The fitting effects and prediction performances of the established models in this study were evaluated through root mean squared error (RMSE) and mean absolute error (MAE).

**Results:**

In Chongqing, 265,824 varicella cases were reported during 2014–2023, the annual average reported incidence rate is 85.99/100,000. The incidence of varicella initially increased and then fluctuated with a downward trend, showing clear seasonality. The peak incidence periods occurred in May–June and October–December each year. The average incidence rates for males and females were 88.92/100,000 and 80.94/100,000, respectively. Children under 15 years old, particularly school-aged children and students, represented the main affected population. The annual incidence rates across districts ranged from 26.90/100,000 to 145.76/100,000. The global spatial autocorrelation analysis indicate that the varicella incidence rate in Chongqing does not exhibit spatial autocorrelation in each year, while the local spatial autocorrelation analysis identified “hotspot” areas primarily concentrated in the main urban metropolitan area. Among the three prediction models based on the monthly incidence rate of varicella from January 2023 to December 2023, LSTM model has the best prediction performance, with RMSE and MAE of 1.52 and 1.19, respectively. The RMSE and MAE of the SARIMA model are 1.91 and 1.49, respectively, while the RMSE and MAE of the SARIMA-LSTM model are 1.99 and 1.47, respectively.

**Conclusion:**

Sustained and effective measures need to be adopted to better curb the spread and prevalence of varicella, particularly among children and adolescents, as well as in the central urban areas and other high-incidence regions. The LSTM model can effectively predict varicella incidence trends, providing scientific evidence to assist relevant authorities in making decisions regarding varicella prevention and control.

## 1 Introduction

Varicella is primarily transmitted via aerosol droplets or direct mucosal contact, classified as an acute contagious respiratory illness with high contagiosity and low fatality rate. The primary clinical features of varicella include the sequential and rapid development of macules, papules, vesicles, and scabs on the skin and mucous membranes. In addition, patients often present with fever and other symptoms (1). Severe complications may occur among a minority of varicella patients, such as septicemia and visceral hemorrhage, and varicella itself can be fatal in some severe cases (2). According to the World Health Organization (WHO), approximately 4.2 million cases of varicella worldwide develop severe complications each year, resulting in nearly 4,200 related deaths (3). Varicella is highly contagious, spreading quickly, making it prone to outbreaks in collective institutions, particularly in childcare facilities, kindergartens, primary and secondary schools, which can significantly impact students’ physical health and the normal teaching work (4). Surveillance data indicates that the reported incidence of varicella in China increased more than 20 times from 2005 to 2019, rising from 3.17 per 100,000 in 2005 to 70.14 per 100,000 in 2019, with a cumulative total of over 6.4 million reported cases. Among them, the average annual reported incidence rate in each province ranged from 12.64 per 100,000 to 78.30 per 100,000, mainly concentrated in the northwest and southwest regions of China from 2005 to 2015; from 2016 to 2019, the incidence ranged from 19.66 per 100,000 to 117.17 per 100,000, mainly due to the rapid increase of incidence rate in east and south regions of China. Provinces such as Jiangsu, Chongqing, Anhui, Shanghai and Guangdong have ranked among the top in the country (5). According to the data of 2019 National Infectious Disease Monitoring System, varicella has become the third largest preventable infectious disease in China, with 981,700 reported cases, second only to tuberculosis and influenza (6), the direct and indirect disease burden associated with these cases cannot be underestimated.

There is no specific medicine for treating varicella, although the varicella vaccine is a preventive vaccine, apart from getting vaccinated, there are almost no other safer and more effective measures to prevent the occurrence of varicella (7). The WHO has recommended that countries with a significant public health burden of varicella carry out routine immunization for children (8). Despite its widespread use in China since 2000 (9), it is still a self-paid vaccine and does not belong to the national immunization program vaccines that can be administered for free (10). A sampling investigation on the national vaccination situation of varicella vaccine conducted in 2020 showed that the proportion of children aged 1–14 who had received 1 dose of vaccine is 52.72%, and the proportion of children who had received 2 dose of vaccine is only 11.43%, indicating an unsatisfactory vaccination rate (11). Thus, controlling and preventing varicella remains an urgent issue for China. Chongqing is the only municipality in the southwest region of China, and the permanent resident population of the city is 31.9143 million at the end of 2023. The climate characteristics of hot and rainy summers and mild and dry winters make the spread and prevalence of varicella in Chongqing have certain local characteristics. The ongoing spread and increasing prevalence of varicella in Chongqing have emerged as a critical public health concern currently (12). Thus it is essential to characterize the epidemiology of varicella in Chongqing, systematically revealing its spatiotemporal distribution patterns and epidemic trends.

The spread and prevalence trend of infectious diseases can be predicted in advance through early prediction, it will provide crucial guidance for epidemic containment. Currently, infectious disease prediction models primarily include linear models, nonlinear models, and ensemble models (13, 14). SARIMA model is a commonly used and classic model for infectious disease forecasting. Due to its simple structure, ease of implementation, and high predictive accuracy, it is widely applied in forecasting diseases with periodic or seasonal trends. Although SARIMA can effectively model the linear components in time sequence, it cannot handle complex nonlinear dependencies (15), and is not adept at dealing with data with unstable long-term lag characteristics (16). However, the occurrence of infectious diseases is influenced by numerous uncertain factors, often exhibiting nonlinear characteristics, with linear models frequently failing to reflect the true situation (17). As representatives of deep learning methods, LSTM model is not only effective in handling and predicting time series data with long-term lags, but it also possess strong nonlinear mapping capabilities (18). The hybrid models, by combining the advantages of multiple models, can help overcome the limitations of individual models to a certain extent, thereby improving the accuracy and stability of predictions (19). However, the epidemic process of infectious diseases has significant regional heterogeneity. Although there have been studies predicting the incidence trend of varicella in time series, existing work is mostly focused on the national level, some provinces or other cities. There is relatively little research on the construction and comparison of prediction models for the epidemic trend of varicella in Chongqing, and there is a lack of direct basis and model support for local varicella prevention and control. Directly applying prediction models from other regions often makes it difficult to fully capture the epidemic pattern of the disease in the local area. Therefore, it is necessary to find a suitable prediction model for the incidence trend of varicella in Chongqing and explore the predictive effects of different models.

In conclusion, this research aims to characterize the epidemiology of varicella in Chongqing during 2014–2023, reveal the spatial distribution patterns of varicella, and explore the establishment of appropriate predictive models based on the surveillance data, providing scientific support for early warning of the varicella incidence trend, the formulation and the optimizing of precise varicella preventive strategies.

## 2 Materials and methods

### 2.1 Data resources

From January 2014 to December 2023, all of the reported varicella cases in Chongqing are sourced from the “Information management system for infectious disease reporting” of the Chongqing Center for Disease Control and Prevention, which is a subsystem of the “Information management system for disease prevention and control in China.” The reported cases of varicella include suspected cases, clinically diagnosed cases and laboratory confirmed cases. The diagnostic criteria are based on the 2023 edition of the Varicella Diagnosis and Treatment Plan issued by the National Health Commission of China (20). Anonymous case data for all reported varicella cases were extracted from the system. The monthly incidence (per 100,000) for each year is calculated by taking the quotient of monthly varicella cases divided by the permanent resident population during the same period, then multiplying by 100,000. The permanent resident population data for each district and county during the same period were derived from the Chongqing Statistical Yearbook.

### 2.2 Study area

Chongqing is a municipality situated in the southwestern part of China, covering a total area of 82,400 km^2^. It has diverse landform types, mainly mountainous, and falls under the subtropical monsoon humid climate zone, featuring an early arrival of spring, sweltering summers, rainy autumns, and warm winters. It is also characterized by abundant precipitation, moist air, and concurrent rain and heat during the same season (21). Chongqing comprises 26 districts, 8 counties and 4 autonomous counties, and the whole city is divided into three regions (22): The main urban metropolitan area (including 21 districts and 1 counties: Banan, Beibei, Bishan, Changshou, Dazhu, Dadukou, Fuling, Hechuan, Jiangjin, Jiangbei, Jiulongpo, Nanchuan, Nan’an, Qijiang, Rongchang, Shapingba, Tongnan, Tongliang, Yubei, Yuzhong Yongchuan and Dianjiang) is mainly situated in the central and western regions of Chongqing, with relatively flat terrain, more developed economy and higher urbanization rates compared to the other two regions. The three Gorges Reservoir Area in northeast Chongqing (including 3 districts and 7 counties: Kaizhou, Liangping, Wanzhou, Chengkou, Fengjie, Fengdu, Wushan, Wuxi, Yunyang, and Zhongxian) and the Wuling Mountain area in southeastern Chongqing (including 2 districts and 4 autonomous counties: Qianjiang, Wulong, Youyang, Pengshui, Xiushan, and Shizhu) are predominantly mountainous, with lower urbanization rates and populations largely residing in rural areas.

### 2.3 Methods

#### 2.3.1 Basic epidemiological and statistical analysis

This section characterize the epidemiology of varicella cases in Chongqing during 2014–2023, including the distribution by age, gender, population categories and seasonal characteristics. The Joinpoint regression model is used to conduct the trend analysis of the incidence and the annual percentage change (APC) is also calculated. Calculate the annual incidence rate of varicella in each district and county (/100,000) and create color-coded stratified map of the incidence rate to illustrate the regional distribution of the monthly varicella incidence rates. The varicella incidence rate levels across different regions are classified into six categories, with each category represented by a different color. It should be noted that due to the lack of population structure data in each district and county, the incidence rate is not standardized when calculated, and only rough rates are used for the analysis of the results.

#### 2.3.2 Spatial autocorrelation analysis

Spatial autocorrelation analysis is typically utilized to assess the presence of spatial correlation in the spatial distribution of certain attribute values and quantify its degree. In this study, ArcGIS 10.8.2 software is used to execute both global and local autocorrelation analyses of the spatial distribution of varicella incidence in Chongqing during 2014–2023. The former describes the spatial characteristics of varicella incidence rate across the entire region through the Moran’s *I*, while the latter seeks to identify potentially masked local spatial autocorrelation or explores whether spatial heterogeneity exists (23). If the result of hypothesis test for the global Moran’s *I* (ranging from −1 to +1) is significantly non-zero, it indicates that the varicella incidence rate of the entire region exhibits non-random spatial distribution. Besides, the clustering patterns including hotspot (high-value clustering areas), cold spot (low value clustering areas), or outliers between a local region and its adjacent areas can be identified by local spatial autocorrelation analysis. Among them, hot spot regions are typically areas of high priority for infectious disease containment, and they are the primary focus of the spatial analysis of varicella in this study.

#### 2.3.3 SARIMA model

SARIMA(p, d, q) (P, D, Q)_s_ model is an extended model that adds adaptability to seasonal variations on the basis of the autoregressive integrated moving average (ARIMA) model (19). The six parameters “p”, “d”, “q”, “P”, “D”, and “Q” respectively refer to the non-seasonal and seasonal autoregressive order, the differencing order and the moving average order, and ‘s’ refers to the step size of the time sequence, representing the seasonal variations characteristics.

The SARIMA models were established by adopting the “forecast” and “tseries” packages in R 4.4.2 by the following steps in our study: first, stationarity test. Assess the stationarity of the varicella monthly incidence time sequence by utilizing the Augmented Dickey-Fuller (ADF) unit root test, and the non-stationary original varicella sequence (i.e., the ADF test result is *p* > 0.05) needs to be converted into a stationary sequence through ordinary difference or seasonal difference, the number of differences corresponding to the “d” or “D” values. Second, model identification. Determine and estimate the patterns of the models preliminarily through the autocorrelation function (ACF) charts and partial autocorrelation function (PACF) charts. Third, model selection. Invalid models whose residuals are not white noise sequences as determined by the Ljung-Box test are eliminated, models with the smallest Akaike information criterion (AIC) in this research is selected as the optimal model.

#### 2.3.4 LSTM model

LSTM model is a specialized variant of the recurrent neural network (RNN) that incorporates gating mechanisms (input, forget and output) and cell states (24). These features allow for selective retention or discarding of information along the time dimension, effectively mitigating the problems of gradient vanishing and exploding commonly encountered in RNN (25). This enables the LSTM to better capture long-range dependencies.

The LSTM model is constructed by using the “tensorflow” and “keras” packages in Python 3.13.5 by the following steps in our study: first, Data Preprocessing and Partitioning. The data is standardized and the dataset is divided into a training set, a validation set and a test set. Second, defining the network structure of LSTM. The values of input layer, hidden layer and output layer need to be designed appropriately, with a dropout layer added to adjust the learning rate to reduce the risk of overfitting. Third, model compilation. The loss function, optimizer, and evaluation metrics need to be explicitly specified. Forth, model training. The model is trained by using the training dataset. The parameters of the model are adjusted based on predictive performance on the validation set. The model is retrained on both the training set and the validation set, and then its predictive performance is evaluated on the test set.

#### 2.3.5 SARIMA-LSTM model

We primarily constructs the SARIMA-LSTM model based on a residual sequence combination approach. First, the optimal SARIMA model is used to extract the linear information from the varicella incidence time series, generating the SARIMA model’s predicted values and calculating the residual sequence. Then, the residual sequence generated by the SARIMA model is input into the LSTM model for training to obtain the residual predictions. The final predictive values of SARIMA-LSTM model are the sum of the predictive values of SARIMA model and the residual predictive values of LSTM model.

#### 2.3.6 Measuring for accuracy

The monthly reported varicella incidence rate in Chongqing between January 2014 and December 2022 is used as the training data for models, while the subsequent 12 months of monthly reported varicella incidence rate(between January 2023 and December 2023) served as the data for testing. RMSE and MAE are two indicators selected to evaluate the fitting effects and prediction performances of the established models in this study. The smaller the value for each of these two metrics, the better models’ fitting effects and prediction performances. The equations of the two indicators are as follows:


$$\text{RMSE}=\sqrt{\frac{1}{n}\sum_{i=1}^{n}{\left(y_{i}-{\hat{y}}_{i}\right)}^{2}}$$

$$\mathrm{MAE}=\frac{1}{n}\sum_{i=1}^{n}|y_{i}-{\hat{y}}_{i}|$$

where $y_{i}$ is the actual monthly varicella incidence rate, ${\hat{y}}_{i}$ is the fitted or predicted monthly varicella incidence rate from the model, n is the number of data points in the model that need to be fitted or predicted.

## 3 Results

### 3.1 Epidemiological characteristics of varicella

#### 3.1.1 Time distribution of varicella

**Figure 1:**
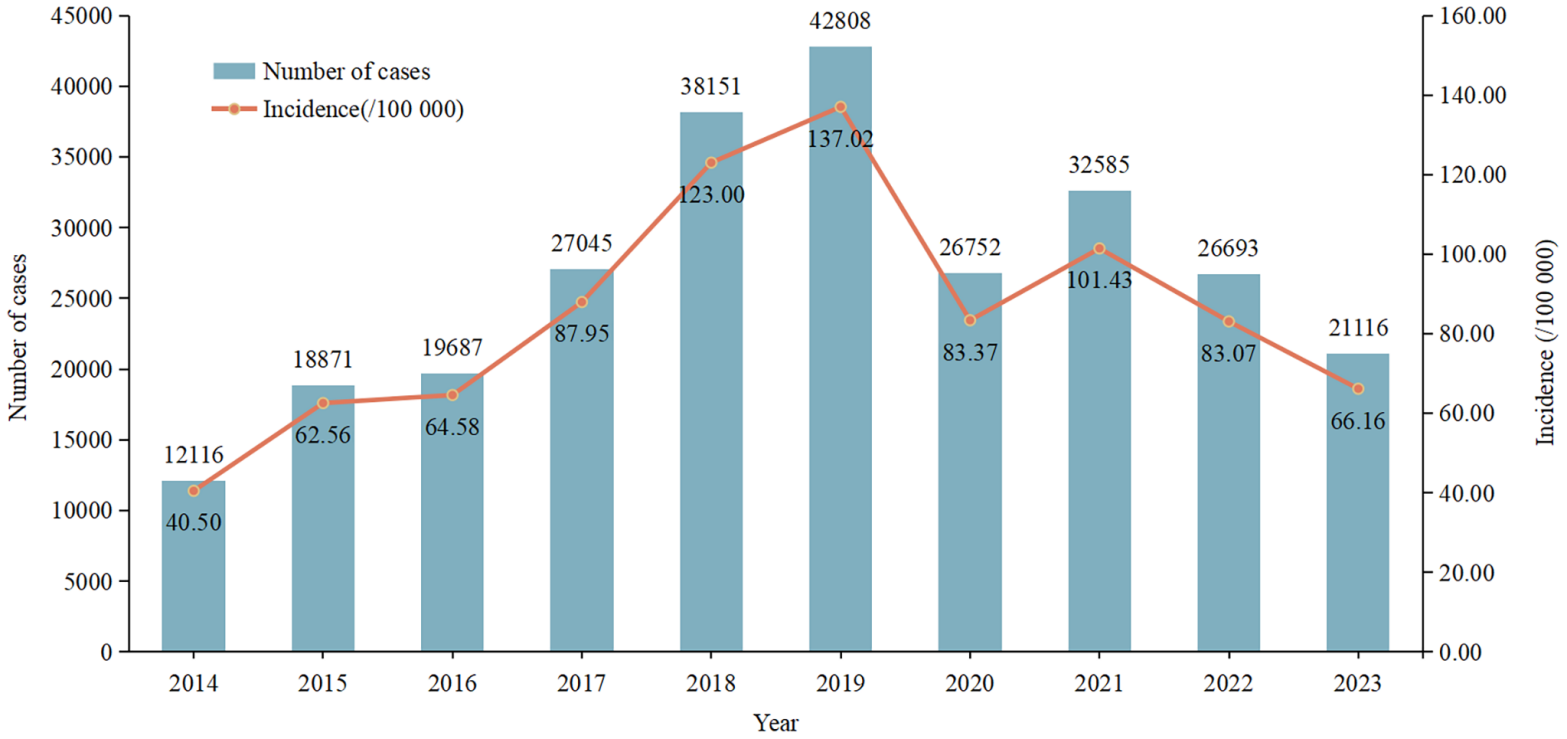
Annual reported cases and incidence rates of varicella in Chongqing, 2014–2023.

**Figure 2:**
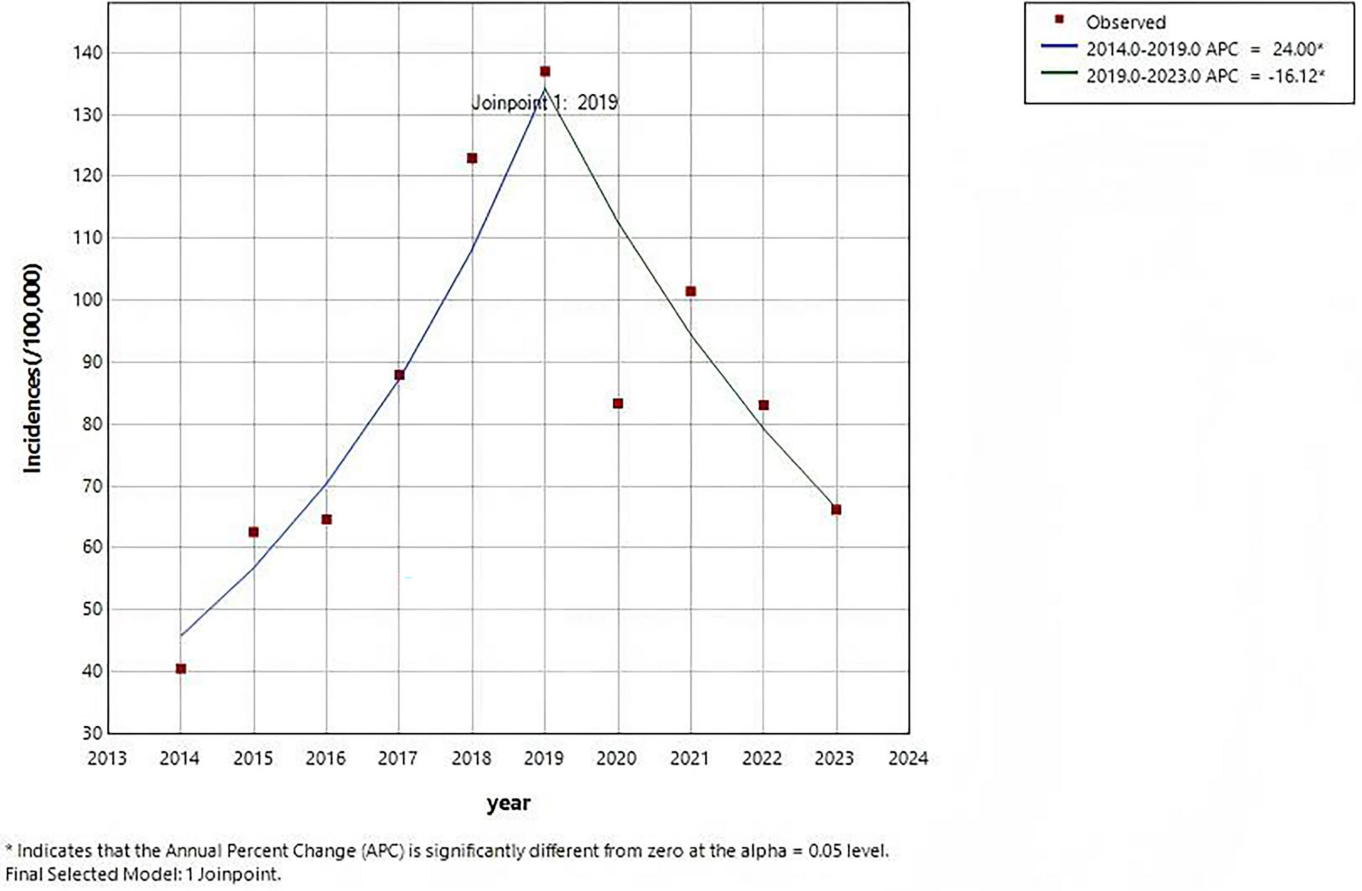
Joinpoint regression analysis of varicella incidences in Chongqing, 2014–2023.

In Chongqing, 265,824 cases of varicella were reported during the period of 2014–2023, with an average annual incidence rate of approximately 85.99/100,000. The annual reported incidence of varicella in 2014 is the lowest, at about 40.50/100,000, while the highest annual reported incidence is recorded in 2019, at approximately 137.02/100,000 (Figure 1). The results of Joinpoint regression model show that the incidence rate of varicella takes 2019 as the turning point, from 2014 to 2019, the reported incidence rate showed an upward trend (APC = 24.00%, *p*<0.001), dropped sharply in 2020, and recovered in 2021. However, from 2019 to 2023, the overall reported incidence has been on a downward trend (APC = −16.12%, *p*<0.001) (Figure 2).

**Figure 3:**
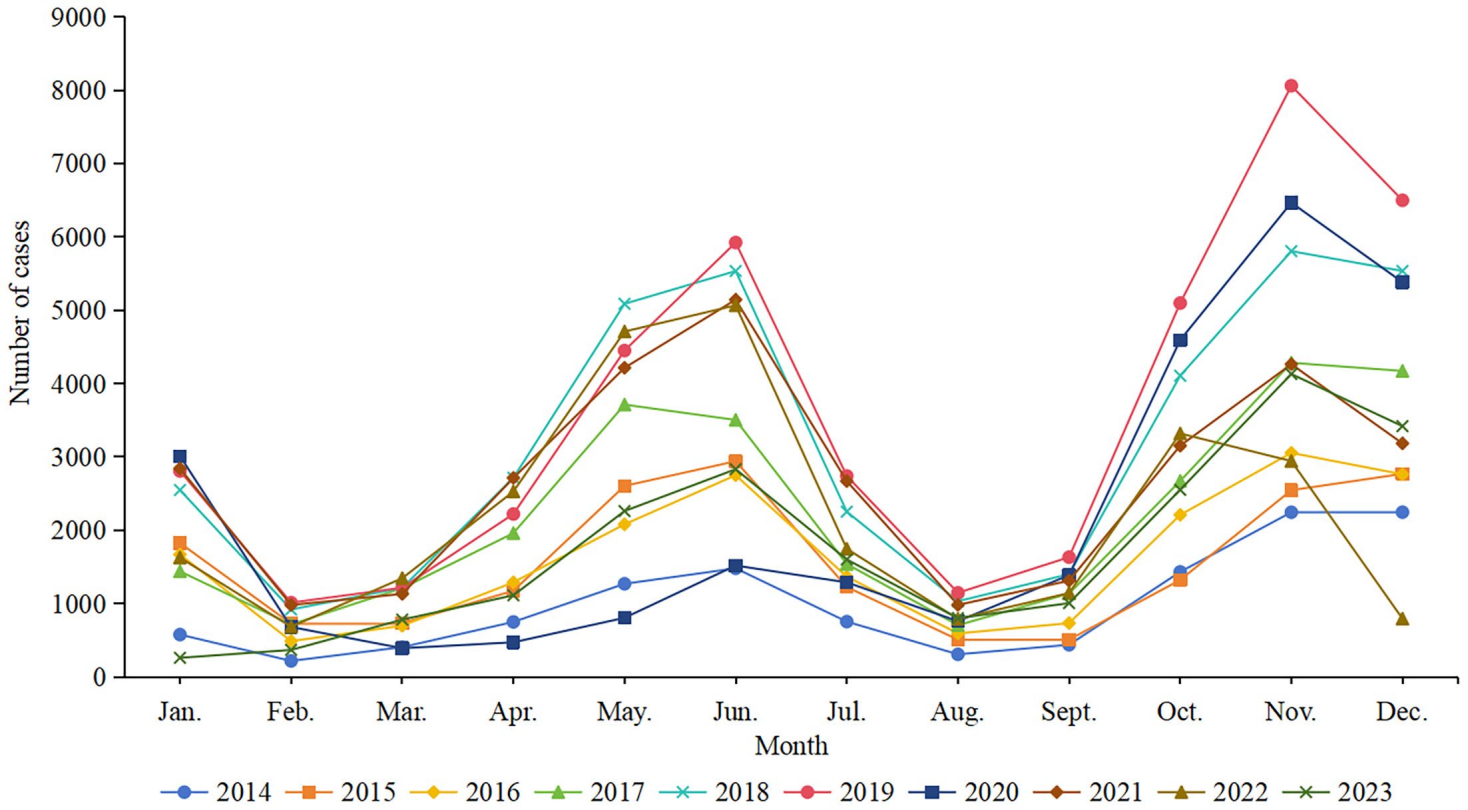
Monthly reported cases of varicella in Chongqing, 2014–2023.

Varicella cases were reported every month from 2014 to 2023 in Chongqing, with a bimodal distribution. The seasonal pattern of varicella incidence each year remained similar, the primary peak occurring during the period from October to December, and the secondary peak occurring during the period from May to June (Figure 3). The reported case numbers for these periods accounted for 41.77% (111,022 cases) and 25.54% (67,890 cases) of the total reported cases, respectively. The number of reported cases in February, March and August, September is relatively low, accounting for 5.98% (15,899 cases) and 6.89% (18,306 cases) of the total reported cases, respectively. The average incidence rate during the peak periods (May, June, October, November and December) is 11.44 per 100,000, and during the trough periods (February, March, August and September), it is 2.73 per 100,000, with a peak-to-valley ratio of 4.19:1.

#### 3.1.2 Demographic distribution of varicella

There were 140,471 reported male cases and 125,353 reported female cases of varicella in Chongqing during 2014–2023. In this decade, the average incidence rates for males and females were 88.92/100,000 and 80.94/100,000 respectively, with a ratio of incidence rates of 1.10:1. Most cases were among individuals aged 15 years and below, with a total of 201,766 cases, accounting for 75.90% of the total cases. In terms of population distribution, school-age children and students were the primary affected groups each year, representing 88.94% of all reported cases (Table 1).

**Table 1:** Demographic characteristics of varicella cases in Chongqing, 2014–2023.

Variables	2014	2015	2016	2017	2018	2019	2020	2021	2022	2023	Total
Age, *n* (%)											
0~	2,114 (17.45)	3,354 (17.77)	3,454 (17.54)	4,494 (16.62)	6,330 (16.59)	6,862 (16.03)	4,012 (15.00)	4,608 (14.14)	3,411 (12.78)	1920 (9.09)	40,559
5~	5,181 (42.76)	8,085 (42.84)	7,498 (38.09)	10,356 (38.29)	14,420 (37.80)	14,635 (34.19)	8,029 (30.01)	9,131 (28.02)	7,891 (29.56)	4,670 (22.12)	89,896
10~	2,247 (18.55)	3,949 (20.93)	4,386 (22.28)	6,609 (24.44)	10,008 (26.23)	11,862 (27.71)	8,026 (30.00)	10,059 (30.87)	7,867 (29.47)	6,298 (29.83)	71,311
15~	1,142 (9.43)	1,489 (7.89)	1962 (9.97)	2,458 (9.09)	3,305 (8.66)	4,541 (10.61)	3,594 (13.43)	5,046 (15.49)	4,515 (16.91)	5,162 (24.45)	33,214
20~	1,090 (9.00)	1,490 (7.90)	1,697 (8.62)	2071 (7.66)	2,530 (6.63)	2,938 (6.86)	1,669 (6.24)	1972 (6.05)	1,598 (5.99)	1,598 (7.57)	18,653
30~	342 (2.82)	504 (2.67)	690 (3.50)	1,057 (3.91)	1,558 (4.08)	1970 (4.60)	1,422 (5.32)	1769 (5.43)	1,411 (5.29)	1,468 (6.95)	12,191
Gender, *n* (%)											
Male	6,383 (52.68)	10,047 (53.24)	10,399 (52.82)	14,192 (52.48)	19,815 (51.94)	22,540 (52.65)	14,007 (52.36)	17,287 (53.05)	14,403 (53.96)	11,398 (53.98)	140,471
Female	5,733 (47.32)	8,824 (46.76)	9,288 (47.18)	12,853 (47.52)	18,336 (48.06)	20,268 (47.35)	12,745 (47.64)	15,298 (46.95)	12,290 (46.04)	9,718 (46.02)	125,353
Population categories, *n* (%)											
School-age children and students	10,698 (88.30)	16,893 (89.52)	17,315 (87.95)	24,046 (88.91)	34,166 (89.55)	38,093 (88.99)	23,874 (89.24)	29,139 (89.42)	23,895 (89.52)	18,315 (86.74)	236,434
Other categories	1,418 (11.70)	1978 (10.48)	2,372 (12.05)	2,999 (11.09)	3,985 (10.45)	4,715 (11.01)	2,878 (10.76)	3,446 (10.58)	2,798 (10.48)	2,801 (13.26)	29,390
Total	12,116	18,871	19,687	27,045	38,151	42,808	26,752	32,585	26,693	21,116	265,824

“Values are presented as *n* (%). Percentages are column percentages: for each year, % = *n* / total cases in that year × 100. The “other categories” in the Population categories include “Professional technical and management personnel,” “Industrial, commercial and service personnel,” “military personnel,” “Agricultural, forestry, animal husbandry and fishery personnel,” “Housekeepers, the unemployed, the jobless and retirees” and “Unknown profession”.

#### 3.1.3 Spatial distribution

**Figure 4:**
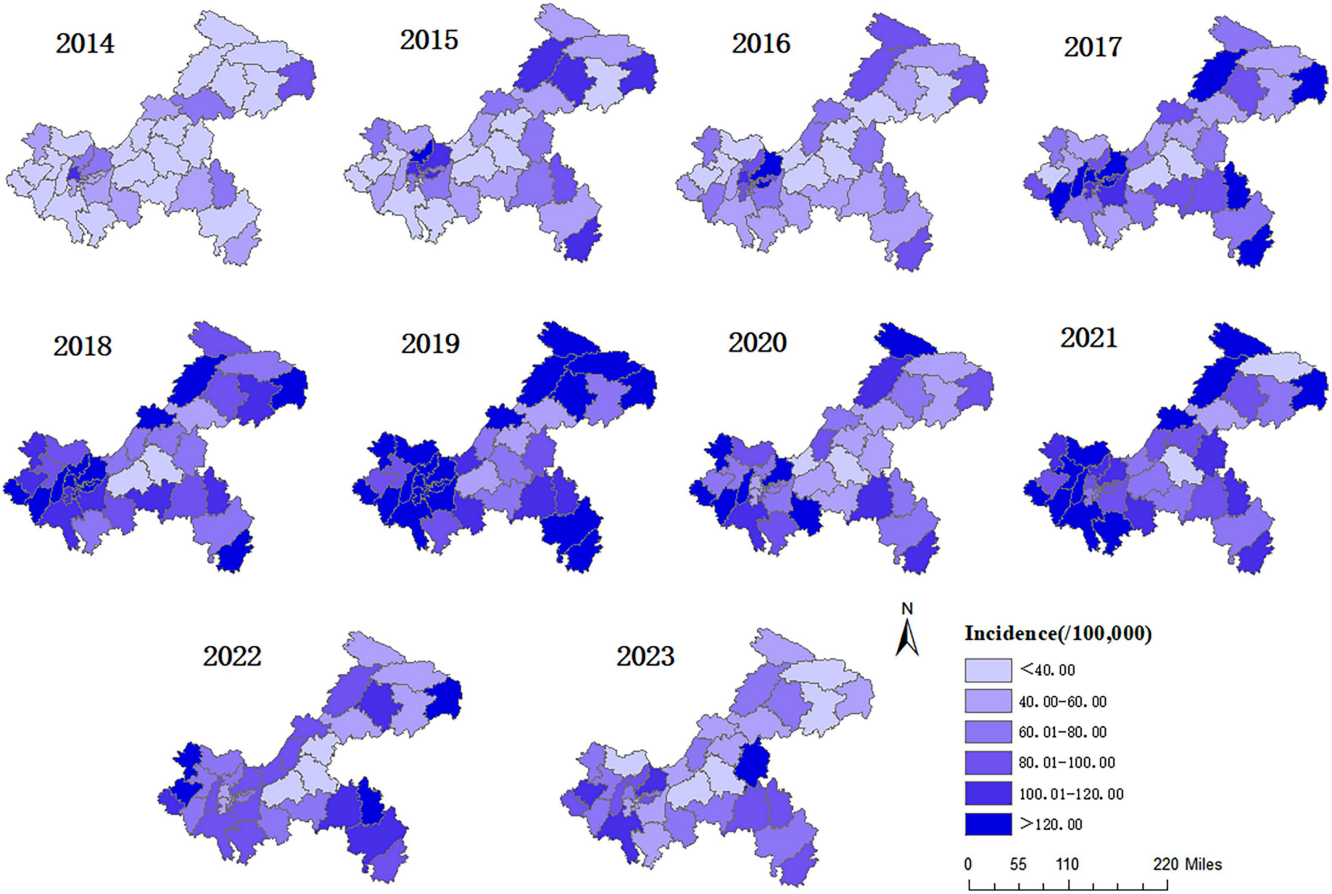
Spatial distribution of varicella incidence in Chongqing, 2014–2023.

Varicella cases were reported annually in all districts and counties during 2014–2023, with the annual incidence ranging from 26.90/100,000 to 145.76/100,000. The top three districts with the highest average annual incidence were Yubei (145.76/100,000), Nan’an (129.53/100,000), and Wushan (125.52/100,000), while the bottom three districts were Fengdu County (26.90/100,000), Fuling District (30.13/100,000), and Zhongxian County (47.10/100,000). As shown in Figure 4, from 2014 to 2019, the number of regions where the incidence of varicella exceeded the average annual incidence rate of the decade from 2014 to 2023 (85.99 per 100,000) has been increasing year by year. In 2014, there were only 2 regions, while in 2019, there were 30 regions. In 2020, the number of regions with an incidence rate exceeding 85.99 per 100,000 decreased to 15. Although this number rebounded to 25 in 2021, it has been decreasing year by year since then. In 2023, the number of regions with an incidence rate exceeding 85.99 per 100,000 was 6.

### 3.2 Spatial autocorrelation analysis

**Figure 5:**
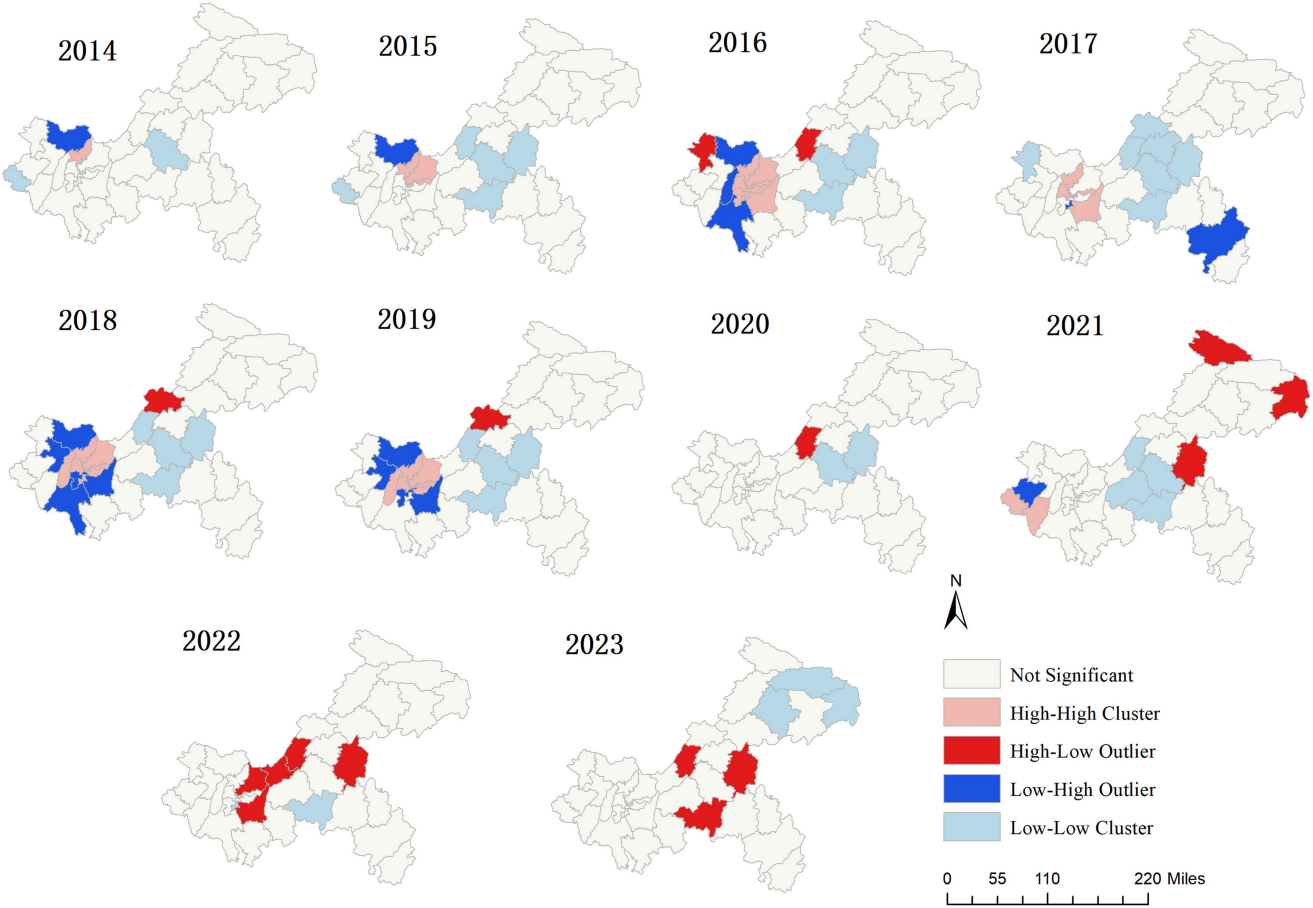
Local spatial autocorrelation cluster maps of varicella in Chongqing, 2014–2023.

The annual global Moran’s *I* of varicella in Chongqing during 2014–2023 are shown in Table 2. However, the *p*-values are not significant, indicating that no stable and consistent global spatial autocorrelation pattern has been observed on a city-wide scale. Local spatial autocorrelation analysis reveals statistically significant local spatial clustering patterns each year (Figure 5). Among them, except for 2020, 2022, and 2023, there are “hotspot” areas (high value and high value regions) in all other years. In 2014, there was 1 hotspot area: Beibei; In 2015, there were 3 hotspot areas: Beibei, Yubei and Jiangbei; In 2016, there were 9 hotspot areas: Beibei, Yubei, Jiangbei, Yuzhong, Nan’an, Dadukou, Banan, Jiulongpo and Shapingba; In 2017, there were 5 hotspot areas: Beibei, Jiangbei, Yuzhong, Banan and Shapingba; In 2018, there were 8 hotspot areas: Beibei, Yubei, Jiangbei, Yuzhong, Nan ‘an, Dadukou, Shapingba and Bishan; In 2019, there were 6 hotspot areas: Beibei, Yubei, Jiangbei, Nan ‘an, Shapingba and Bishan; In 2021, there were 2 hotspot areas: Rongchang and Yongchuan. Although the locations of the four clustering patterns vary each year, the hotspot areas are primarily located in the main urban metropolitan area.

**Table 2:** Global spatial autocorrelation results of varicella in Chongqing, 2014–2023.

Year	*Moran’I*	*Z-*Score	*p*-value
2014	−0.0034	0.2296	0.8184
2015	0.0511	0.9771	0.3285
2016	0.0987	1.2174	0.2234
2017	0.0438	0.6735	0.5006
2018	0.1374	1.6039	0.1087
2019	0.1380	1.5740	0.1155
2020	0.0706	0.4270	0.6694
2021	−0.0122	0.1419	0.8872
2022	−0.0541	−0.2685	0.7883
2023	0.0342	0.5874	0.5569

### 3.3 Monthly incidence prediction model

#### 3.3.1 SARIMA model

**Figure 6:**
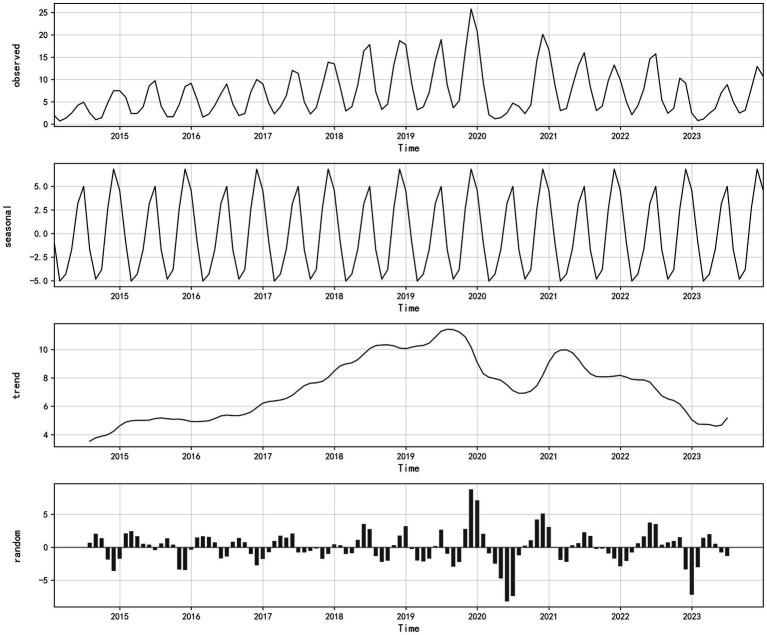
Time series decomposition chart of varicella incidence in Chongqing, 2014–2023.

From the time sequence decomposition chart of the monthly varicella incidence rate, it can be observed that the time series of varicella incidence is decomposed into three components: season, trend and residual. From the decomposition of the seasonal term, it can be known that the incidence shows obvious seasonality, and with a 12-month cycle, the incidence rate of the second peak is significantly higher than that of the first one, which means varicella is commonly found to occur in winter and spring (Figure 6).

**Figure 7:**
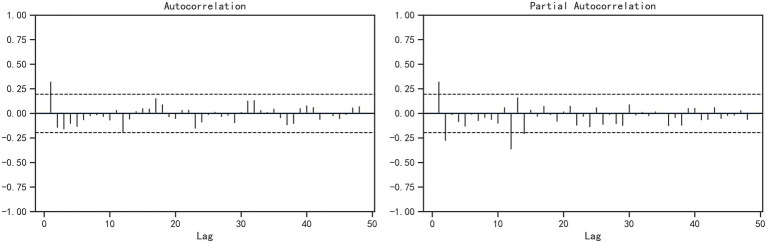
ACF and PACF plots of the stabilized monthly incidence of varicella in Chongqing, 2014–2023.

The stationarity test results show that the time sequence of the monthly reported varicella incidence rate during 2014–2023 is non-stationary (ADF = −2.04, *p* = 0.27). After applying non-seasonal differencing (d = 1) and seasonal differencing (D = 1), a stationary series is obtained (ADF = −3.93, *p* < 0.05). Based on the decomposition of the seasonal term of the aforementioned varicella incidence rate, the model parameter s = 12 can be determined. The ACF and PACF of the differenced series are shown in Figure 7. In the ACF plot, the autocorrelation coefficient at lag 1 is outside the two standard deviation range, while the autocorrelation coefficients of other lags fluctuate within the two standard deviation range and exhibit a slow decay to zero, indicating a cut-off phenomenon, thus the initial value of q is determined to be 0 or 1. The PACF plot shows that the partial autocorrelation coefficients at lags 1 and 2 are outside the two standard deviation range, indicating a tailing phenomenon, thus the initial value of p is determined to be 0, 1 or 2. The values of parameters P and Q are difficult to determine, but according to the prior research experience, they generally do not exceed 2, the optimal model can be selected using a trial method. Therefore, the values of P and Q are 0, 1, or 2 respectively, and they are combined one by one.

**Figure 8:**
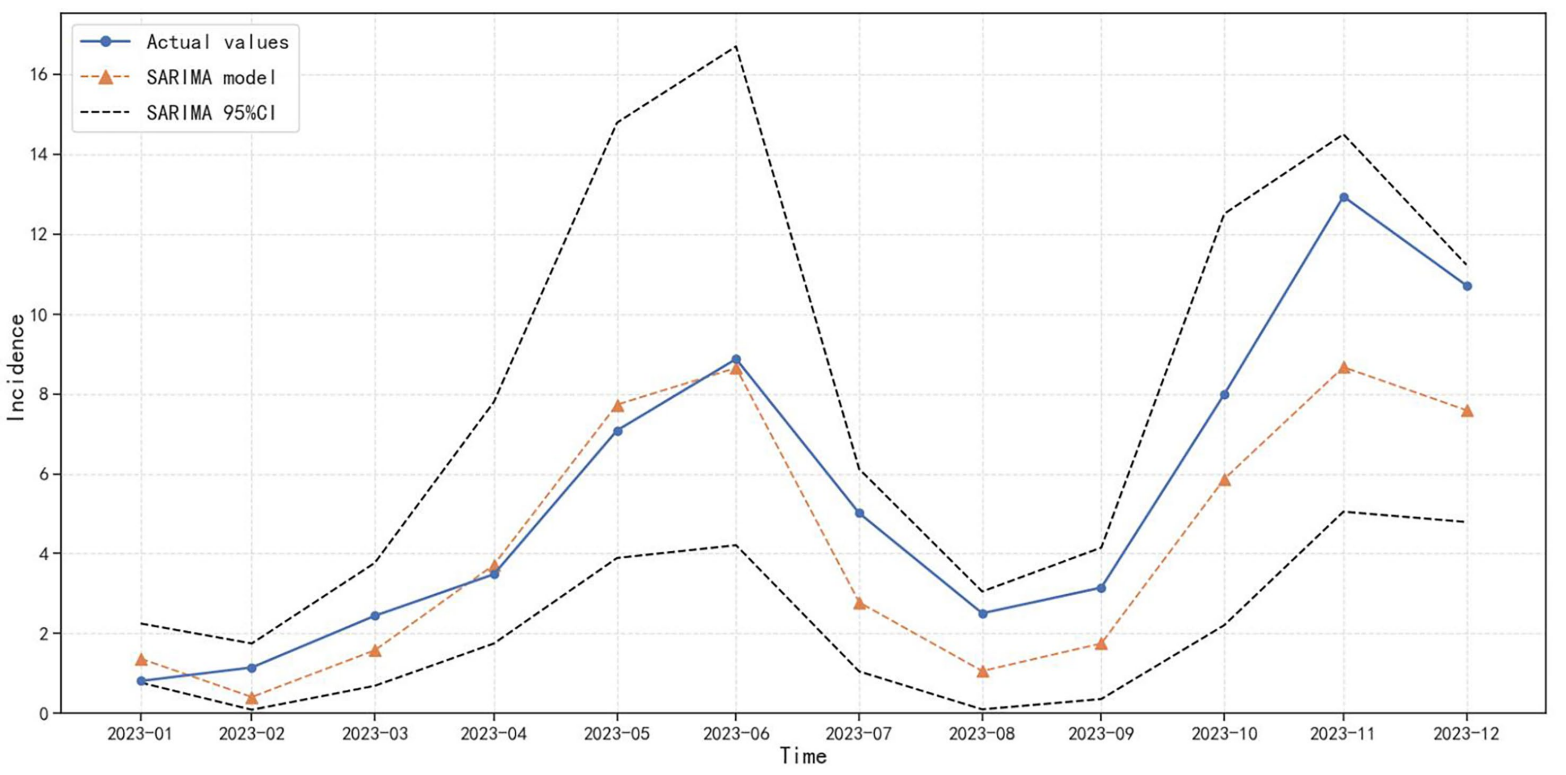
Prediction charts of SARIMA model.

In summary, there are theoretically 2 × 3 × 3 × 3 = 54 parameter options for the SARIMA model. Among the final 54 parameter selections, 31 models are successfully constructed. After the Ljung-Box test, the residual sequences of the remaining 12 SARIMA models are white noise sequences (*p* > 0.05). Based on the principle of smallest AIC and that all model parameters are statistically significant, the SARIMA (1,1,1) (0,1,0)_12_ model is initially selected. The model AIC = −41.53, and the *p*-value of the residual white noise test is 0.25. The test results of each parameter of the model are shown in Table 3. SARIMA (1,1,1) (0,1,0)_12_ model is used to predict the incidence of varicella, as shown in Figure 8, although there are certain differences between the actual values and the predicted values, the actual values in each month all fell within the 95%CI of the predicted values, and the dynamic trend of the predicted values is in good agreement with the actual values. The feasibility and effectiveness of the model are verified, indicating that the model has a good tracking and prediction effect on the future incidence of varicella.

**Table 3:** The parametric test results of the SARIMA (1,1,1) (0,1,0)_12_ model.

Variable	*B*	*SE*	*t*	*p-*value
AR (1)	0.684	0.203	3.368	*p*<0.001
MA (1)	−0.889	0.141	−6.323	*p*<0.001
MA (2)	0.035	0.004	9.718	*p*<0.001

#### 3.3.2 LSTM model and SARIMA-LSTM hybrid model

Given the relatively limited scale of the varicella dataset, in order to determine the model parameters and avoid information leakage from the test set, this study further divided the training set into a validation set (from January 2022 to December 2022), which is only used for parameter tuning of the model. After retraining the final model on both the training set and the validation set, the predictive performance will be reported on the test set (from January 2023 to December 2023).

As this study is a univariate monthly sequence prediction and the size of training sample is relatively small, using a more complex LSTM structure is prone to overfitting and has limited benefits. Thus a single-layer LSTM structure is adopted in this study. From the aforementioned analysis, it can be found that the original sequence of the monthly incidence of varicella has obvious annual periodicity, so the window length of the LSTM model is set to 12, which means the time step is set to 12, and the following one-month is used as output for prediction, which means the number of nodes in the output layer is set to 1. A smaller batch can increase the frequency of parameter updates, which is helpful for stably learning the local variation features of the sequence when the sample size is small, so the batch size is set to 1.

When determining the optimal number of neurons in the hidden layer, this study adopted a strategy of conducting a series of experiments with a power of 2 as the number of neurons in the hidden layer. As shown in Table 4, while keeping other parameters unchanged, when the number of neurons in the hidden layer of the LSTM model is set to 64 and the number of neurons in the hidden layer of the SARIMA-LSTM model is set to 128, the two key evaluation indicators of RMSE and MAE of both reached the lowest level. To ensure that the model fully learns the sequence pattern while controlling the risk of overfitting, the training rounds (epochs) are set to 120, and regularization (Dropout = 0.1) and validation set are combined to monitor the training process, to ensure that the model training converges and does not overfit. The Adam optimizer is adopted and adaptive learning rate with initial value set to 0.001. Finally, after determining the above parameter configuration, the models are constructed to predict the incidence of varicella.

**Table 4:** Comparison of prediction errors of models with different numbers of neurons.

Model	Hidden neurons	RMSE	MAE
LSTM	2	2.88	2.30
	4	3.39	2.72
	8	2.94	2.27
	16	1.80	1.42
	32	1.92	1.58
	64	1.57	1.33
	128	2.04	1.71
SARIMA-LSTM	2	3.40	2.34
	4	3.31	2.09
	8	3.16	2.19
	16	2.41	1.70
	32	2.40	1.69
	64	2.16	1.66
	128	2.05	1.53

#### 3.3.3 Comparisons of the forecasting performance

**Figure 9:**
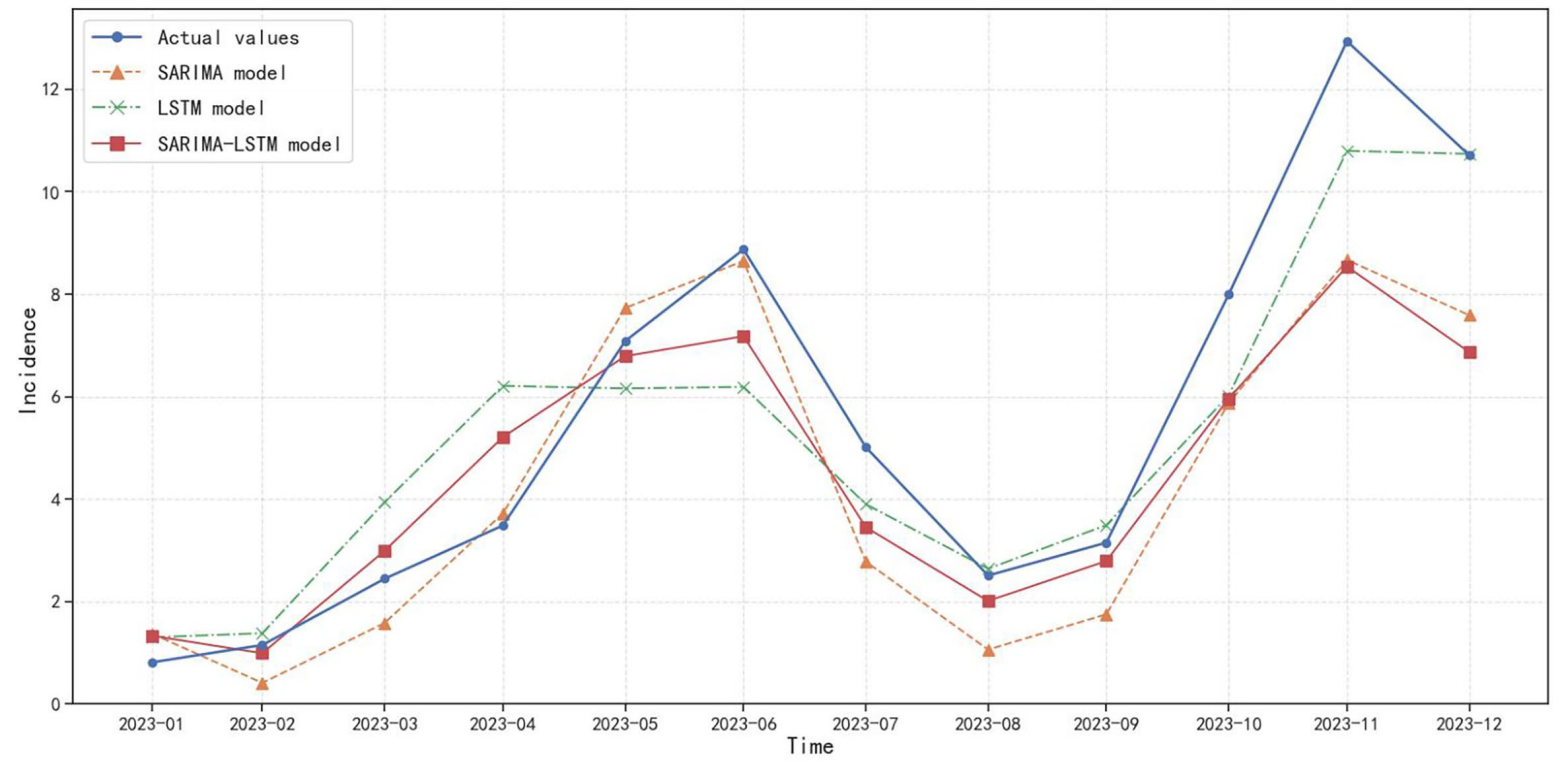
Prediction charts of SARIMA, LSTM, and SARIMA-LSTM models.

Overall, all three prediction models accurately forecast the varicella incidence trend in Chongqing from January 2023 to December 2023 (Figure 9), indicating that the established models in our research provide generally reliable assessments of the varicella epidemic trend. However, from the perspective of the three model evaluation indicators, as shown in Table 5, the RMSE and MAE of the SARIMA model are 1.91 and 1.49 respectively; the RMSE and MAE of the SARIMA-LSTM model are 1.99 and 1.47 respectively, and there is not much difference in their prediction performance. The RMSE and MAE of the LSTM model in the test set are the smallest, which are 1.52 and 1.19, respectively. Compared to the SARIMA model, the RMSE of the LSTM model decreased by 20.42%, and the MAE decreased by 20.13%. Compared to the SARIMA-LSTM model, the RMSE of the LSTM model decreased by 23.62%, and the MAE decreased by 19.05%. This suggests that the LSTM model provides better predictions of varicella incidence trends in Chongqing.

**Table 5:** Comparison of fitting and prediction effects of SARIMA, LSTM, and SARIMA-LSTM models.

Month	Actual values (1/100,000)	SARIM A model (1/100,000)	LSTM model (1/100,000)	SARIMA-LSTM hybrid model (1/100,000)
2023–01	0.81	1.37	1.30	1.33
2023–02	1.15	0.41	1.38	0.99
2023–03	2.44	1.58	3.94	2.99
2023–04	3.48	3.72	6.21	5.21
2023–05	7.09	7.73	6.16	6.79
2023–06	8.87	8.65	6.19	7.18
2023–07	5.01	2.78	3.90	3.45
2023–08	2.51	1.06	2.64	2.01
2023–09	3.15	1.75	3.49	2.79
2023–10	7.99	5.87	6.02	5.96
2023–11	12.94	8.67	10.80	8.54
2023–12	10.71	7.59	10.74	6.87

Indicators	Training set	Test set	Training set	Test set	Training set	Test set
RMSE	1.79	1.91	1.42	1.52	1.58	1.99
MAE	1.35	1.49	1.03	1.19	1.26	1.47

## 4 Discussion

The varicella cases in this study are all sourced from the “Information management system for infectious disease reporting” of the Chongqing Center for Disease Control and Prevention. Although China has issued standardized diagnosis and treatment plan for varicella, uncertainties may still exist in real-world diagnosis and monitoring. For example, before the appearance of the rash, varicella may present with transient low-grade fever, fatigue, and other prodromal symptoms, but the clinical manifestations during this stage lack specificity, which may lead to difficulties in early identification (26); breakthrough infection cases may present with atypical clinical manifestations such as a smaller number of rashes and predominance of maculopapules; there is a certain clinical similarity between varicella and various rash-inducing diseases such as hand, foot, and mouth disease, herpes simplex, and impetigo in cases of mild or focal rashes. All of the above situations increase the difficulty of clinical identification and differential diagnosis, and may affect the sensitivity of case reporting (27). In addition, laboratory confirmation is often used in surveillance practice for severe, atypical cases or outbreak investigations. For cases with co-infection or severe complications, pathogen detection (such as DFA or PCR) or imaging evaluation (such as chest X-ray or MRI) is usually required to assist in differential diagnosis (28). However, routine cases are mainly clinically diagnosed, so the reported incidence rate may also be affected to some extent by diagnostic practice and testing accessibility (29).

During the period of 2014–2023, the gradual increase in reported varicella cases in Chongqing may be related to the policy implemented in 2014 by the Chongqing Health Administration, which regards varicella as Class C notifiable infectious disease for surveillance and reporting (Class C notifiable infectious diseases in China’s classification system for notifiable infectious diseases refer to common or sporadic infectious diseases with relatively weak infectivity, low fatality rates, limited epidemic ranges, and requiring routine monitoring and management) (30). During the diagnosis and treatment process, if the first-time doctor discovers varicella patients, they need to fill out the infectious disease report card according to relevant requirements and report directly online within 24 h. Medical institutions that do not have the conditions for direct online reporting should promptly report to the local township health center, urban community health service center, or county-level disease prevention and control institution, and send the infectious disease report card to the reporting agency within 24 h. The standardization of varicella case reporting was strengthened, monitoring quality was improved, and new cases in various regions could be registered quickly and efficiently in the surveillance system after the policy was enacted. In 2020, the varicella reported incidence rate in Chongqing sharply declined, followed by a fluctuating downward trend, which is similar to the situation in other provinces and cities in China during the same period, such as Sichuan Province (31), Shaanxi Province (32) and Nanjing (33). The primary reason for the phenomenon could be the implementation of containment measures such as disinfection of public places, frequent hand washing, mask-wearing, and quarantine at home during the COVID-19 pandemic. These measures reduced group activities, with schools switching to online teaching, thus decreasing student interactions (34). This led to a reduction in the occurrence of a series of respiratory infectious diseases, including varicella (35, 36). In addition, the utilization of medical services declined during the epidemic, and some mild varicella patients may reduce their visits or self-isolate at home. The monitoring data of this study depends on the initiative of medical institutions to report, and the reported incidence rate of varicella during this period may also underestimate the actual situation to some extent. It is noteworthy that an increasing number of studies suggest that long COVID infection can affect the human immune system, thereby influencing the body’s susceptibility to other pathogens (37, 38). Therefore, the fluctuating changes in the reported incidence of varicella in Chongqing after 2020 may also be influenced by multiple factors such as changes in the population’s immune status. Except for 2020, the seasonal distribution characteristics of varicella incidence in Chongqing each year are generally similar. This bimodal distribution pattern is consistent with those in other provinces and cities in China (31–33), but differs from regions such as Thailand (39), Japan (40), Denmark, and Finland (41). The differences in seasonal patterns across regions may be related to the geographical location, climate characteristics, population density, and vaccination status of different areas (42–44).

The majority of reported varicella cases in this study occurring in individuals aged 15 years and below, corresponding to the primary population group for varicella (school-age children and students). The main reason for this is that children’s and adolescents’ immune systems are not fully developed, making them more susceptible to varicella-zoster virus (VZV). Environments such as kindergartens and schools, which are dense and enclosed, increase the likelihood of virus transmission. Additionally, school-age children and students are active, have a broad range of activities, and may not have fully developed hygiene habits, significantly increasing their chances of exposure to VZV. Since there are no specific medications for treating varicella, vaccination is considered the most effective and direct method for controlling the incidence and spread of the disease (45). However, due to the different situations in various regions of China, most regions have not yet incorporated varicella vaccines into immunization program (46), and the vaccination rate among children in some areas of Chongqing is relatively low (47). Therefore, relevant authorities need to focus on increasing awareness of varicella vaccination among students, parents, and teachers, strengthening monitoring of varicella and other infectious diseases in schools and daycare institutions, and enhancing the promotion of health education, particularly regarding hygiene, to raise public awareness of varicella prevention.

The results of the annual average incidence rate of varicella in all regions of Chongqing indicate that there have been regions in the main urban areas where the incidence rate has exceeded the annual average incidence rate of varicella in this study over the years. Although the results of global spatial autocorrelation analysis indicate that no stable and consistent global spatial autocorrelation pattern is observed for the reported incidence of varicella in Chongqing on a city-wide scale, since the global Moran’s *I* reflects the overall intensity of the spatial-dependent varicella incidence on a city-wide scale, when the significant aggregation is limited to only a few districts and counties, and the remaining areas are approximately randomly distributed, The global Moran’s *I* may not be significant. In contrast, LISA is a local statistic that can identify significant clusters or outliers between a few districts and counties and their neighboring areas. The results of the local spatial autocorrelation analysis in our study also indicate that the hotspot areas primarily located in the main urban metropolitan area of Chongqing. This is likely due to the high population density and frequent population movement in these areas, which increases the likelihood of virus transmission. Besides, since varicella vaccination is self-paid in most regions and the vaccination coverage in the main urban metropolitan area is not high, most people only receive the first dose of the vaccine (47, 48). Therefore, it is recommended to optimize the vaccination strategy to enhance immunization coverage in regions with high incidence, such as promoting the implementation of policies that benefit the people for varicella vaccination, providing convenient vaccination services for different populations, or strengthening the mechanism for detecting and supplementing missed doses, providing precise vaccination for groups who have not completed two doses of vaccination to achieve a more robust protective immunity against varicella (49, 50), these interventions could be effective to curb the spread of varicella and reduce its prevalence in the population.

SARIMA, LSTM, and SARIMA-LSTM hybrid models have been widely applied in the prediction of various infectious diseases with similar epidemiological mechanisms to varicella, such as hand, foot, and mouth disease (HFMD) (51–53), tuberculosis (54–56), and influenza (57–59), achieving satisfactory results. The SARIMA model can effectively capture the linear features and seasonal dynamics of the data, enabling it to extract linear information from the data (15). The LSTM model selectively retains or discards information along the time dimension through its gating mechanisms, which can solve the problems of long-term dependence and vanishing gradients, it is particularly suitable for data with clear periodicity and large fluctuations (18). Combining these two models can leverage their respective advantages to some extent (60). However, since each model is applicable to specific diseases and data types, no single statistical technique can ideally fit every disease (61). From the perspective of fitting effects and prediction performances, the LSTM model in this study shows higher prediction accuracy in short-term prediction (12 months) of the monthly incidence rate of varicella in Chongqing, and can more clearly reflect the varicella epidemic trend. However, it is still necessary to further evaluate the robustness and generalization of the model through prospective validation or rolling window evaluation in the future.

## 5 Limitations

In addition to the above research findings, some limitations in this research also need to be taken seriously. Firstly, all of the reported varicella cases in Chongqing during 2014–2023 were sourced from the surveillance management system, where the data relies on passive reporting. Missing or misreported cases may affect the accuracy of the analysis. Additionally, for asymptomatic carriers and pathogen carriers, who are difficult to identify, the study results may underestimate the actual transmission risk. Secondly, due to the lack of data such as the vaccination coverage and the situation of immunization gaps in various regions, this study did not conduct a more detailed exploration of the possible mechanisms when analyzing the prevalence of varicella. Thirdly, the prediction model in this study is a univariate monthly series prediction model. Limited by the scale of the dataset, the model structure is relatively simple. Numerous scientific researches have indicated that the spread and prevalence of varicellla can be affected by various factors such as climate, environment, and vaccination status (62), whereas this study only take inherent characteristics of the varicella incidence rate time sequence into considered when establishing the models, and did not include covariates such as meteorology, environment, or socio-economic conditions to construct the model. In our future research, in addition to expanding the length of the time series and introducing more exogenous variables, more advanced modeling methods or frameworks can also be used to attempt to construct other predictive models to enhance the accuracy and usability of the model in public health practice.

## 6 Conclusion

The varicella incidence rate in Chongqing has shown an increasing trend since 2014, with a sharp decline in 2020, followed by a fluctuating downward trend. The seasonal characteristics are evident, and high-risk clusters are mainly concentrated in the main urban districts. Children and students under the age of 15 are the primary groups affected by varicella. The LSTM model is applicable to the short-term prediction of the monthly varicella incidence rate in Chongqing and can serve as a reference model for predicting varicella incidence trends. To better curb the spread and prevalence of varicella, it is necessary to strengthen vaccination efforts, school-based surveillance, and public health education in the main urban areas of Chongqing, while integrating prediction models to dynamically track varicella incidence trends and adopting multiple strategies to reduce the transmission risk.

## Data Availability

The original contributions presented in the study are included in the article/supplementary material, further inquiries can be directed to the corresponding author.
